# Electrochemical Nanolithography on Silicon: An Easy and Scalable Method to Control Pore Formation at the Nanoscale

**DOI:** 10.3390/ma12182891

**Published:** 2019-09-07

**Authors:** Elisa Pinna, Mehran Mehrabanian, Eugenio Redolfi Riva, Eleonora Cara, Giulia Aprile, Luca Boarino, Guido Mula

**Affiliations:** 1 PoroSiLab, Dipartimento di Fisica, Università degli Studi di Cagliari, 09042 Monserrato, Italy; 2 Istituto Officina dei Materiali CNR-IOM, Sezione di Cagliari, 09042 Monserrato, Italy; 3 Istituto Nazionale di Ricerca Metrologica (INRiM), 10135 Torino, Italy

**Keywords:** porous silicon, nanolithography, wet electrochemical etching

## Abstract

Lithography on a sub-100 nm scale is beyond the diffraction limits of standard optical lithography but is nonetheless a key step in many modern technological applications. At this length scale, there are several possible approaches that require either the preliminary surface deposition of materials or the use of expensive and time-consuming techniques. In our approach, we demonstrate a simple process, easily scalable to large surfaces, where the surface patterning that controls pore formation on highly doped silicon wafers is obtained by an electrochemical process. This method joins the advantages of the low cost of an electrochemical approach with its immediate scalability to large wafers.

## 1. Introduction

Lithography is a very powerful method to obtain controlled structures on samples [1,2,3,4] and it has been fruitfully used in the fabrication of electronic devices [5]. The need to obtain patterns with decreasing sizes at first directed the investigations towards the diffraction limit of standard optical lithography [5], whose limit is of about 100 nm, and then to the development of more sophisticated techniques when the desired length scale was below that limit. Methods to achieve sub-100 nm lithography are, for instance, electron beam lithography (EBL) [6] or focused ion beam lithography [7]. These techniques are extremely powerful in terms of spatial resolution, precision, and reproducibility [6,8], but require highly expensive instrumentation and are excessively time-consuming for large surface applications [9]. Possible alternatives to electron or ion lithography come from the use of polymeric nanoparticles [10,11] or block copolymers [10,12] to fabricate regular arrays. These solutions have the remarkable advantage of being readily available for large surfaces. They are often used in combination with metal deposition followed by metal-assisted chemical etching (MACE) [13] or with reactive ion etching (RIE) [14]. RIE has the disadvantage that the maximum depth achievable is limited to the same dimension of the mask openings [15,16], while MACE presents limitations in controlling the etching depth [17,18]. Other electrochemical techniques [19,20] make use of nanoelectrodes to achieve a high precision on the surface control but are still techniques that require very specific electrodes, that need to be replaced if the lithographic requirements change, and are obviously hardly scalable to large surfaces. Other methods [21] do not make use of specific electrodes but require the deposition of an aluminum layer (in this case on titanium layer) for the formation of a lithographic patterning thanks to the porosification of the alumina layer on the surface. In this latter case, the proposed technique requires the use of a preliminary fabrication of an alumina layer before the lithographic process. This is obviously less simple than an electrochemical process that starts on the pristine sample surface, and, moreover, it is not straightforward that this method can be used to obtain electrochemical lithography on Si surface.

The pore formation process in a Si wafer [22] starts on the pristine surface on random sites. This happens since the pore formation is activated at the surface irregularities unavoidably present on any surface, however polished. The irregularities modify locally the electric field and then generate a local charge accumulation that eventually leads to the initiation of the etching process. The fact that the pores start on irregularities is the basis of standard lithographic processes in Si, where the generation of tailored surface irregularities allows for the control of the pore distribution, density, and shape. This is the case, for instance, for the fabrication of macropores [23], and is the same idea behind the process we propose here, the electrochemical nanolithography (ENL), as will be described in what follows.

ENL is an easy and cost-effective technique, apt to control the distribution order and the density of the pore formation at the nanoscale thanks to a process that leaves indentations onto the sample surface that will act as seeds for the formation process of new pores. A patent application has been submitted for this process [24]. The ENL process essentially governs the position and amount of the surface irregularities, while leaving the final shape and size of the pores in the final porous layer, the one to be formed after ENL, at the free choice of the experimenters. This technique is based only on low-cost chemical and electrochemical processes that can be applied to small as well as large-scale samples, requiring only the control of the homogeneity of the solution and of the electric field on the sample surface during the electrochemical etching process. The proposed process is based on the fabrication of a sacrificial double layer (SDL) of porous silicon (PSi) that, after the formation process, is chemically removed taking care to leave the pores’ bottom still on the surface. These residual pores will leave surface indentations acting as nucleation loci for the formation of a third porous layer with independent electrochemical parameters. Hereafter, we will refer to those indentations as “seeds”. The two different porous layers are necessary in order to separately control the pore density and the seed distribution order. In fact, the ENL process allows to obtain indentations having the same density of seeds (and then of the new porous layer) with a different surface arrangement or, vice versa, the same kind of ordering of the seeds but different densities. 

## 2. Materials and Methods 

Porous Si samples were fabricated starting from highly *n*-doped crystalline Si substrates from Sil’tronix (Archamps, France), with 15–18 × 10^−3^ Ω resistivity range. The electrochemical solutions were prepared using HF/H_2_O/EtOH solutions with various relative concentrations. The stabilized current for the porous layers’ formation was given by a PARSTAT 2273 Potentiostat from Princeton Applied Research (Oak Ridge, TN, USA) and from a Keithley SMU 2450 SourceMeter (Beaverton, OR, USA). The electrochemical etching processes were carried out in PVC cells, as described in [25]. The chemical etch for the removal of SDL to obtain the ENL seeds was obtained using aqueous NaOH 1M solution. 

The morphological characterization of the samples was carried out by scanning electron microscopy (SEM) using a field emission gun SEM (FEI Inspect F, FEI, Hillsboro, OR, USA). The SEM micrographs were acquired in top-view and cross-section mode at a fixed working distance of 10 mm. The accelerating voltage was set to 20 kV with a spot size of 3.5.

The fast Fourier transform of SEM images was obtained by using ImageJ software (version 1.52n 22).

### Formation and Role of the Sacrificial Double Layer (SDL)

**Figure 1:**
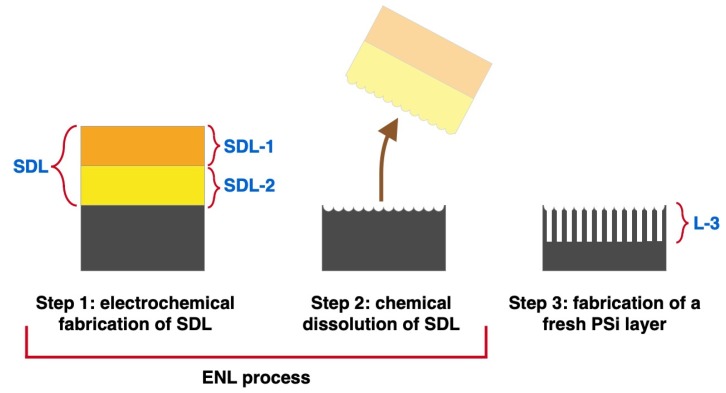
Schematic of the fabrication of a controlled porous layer using the electrochemical nanolithography (ENL) process.

The main goal of ENL is to control the pore density, surface repartition, and size homogeneity in a third layer that will be fabricated after the SDL dissolution, that is after the completion of the ENL process. The schematic of the process leading to the fabrication of a controlled porous layer using the ENL approach is depicted in Figure 1. 

The first step of ENL is the fabrication of the SDL (Figure 1, left), composed of two layers that will be identified as SDL-1 and SDL-2 throughout this work. SDL-1 is the first layer formed, therefore, the one starting at the sample’s surface, while SDL-2 is the second layer formed. The pore formation in SDL-1, since it starts on a polished flat surface, starts on random sites at the surface irregularities, as described earlier. The two layers are evidenced in Figure 1, left. The second step is the chemical removal of the SDL (Figure 1, center), leaving a modified surface whose indentations act as seeds for the formation of new pores. This second step ends the ENL process and leaves the sample ready for the fabrication of a new controlled porous layer, indicated as L-3, as the third step (Figure 1, right).

In ENL, SDL-1 controls the density of pores, since the formation of SDL-2 starts from the bottom of the SDL-1 pores and, therefore, is expected to exhibit a pore density tightly related to that of SDL-1. As a consequence, at the end of the ENL process, that is after the SDL removal, the SDL-1 pore density is expected to be transferred to the density of the seeds left on the surface. This behavior will be discussed in detail and demonstrated in the next paragraphs.

**Figure 2:**
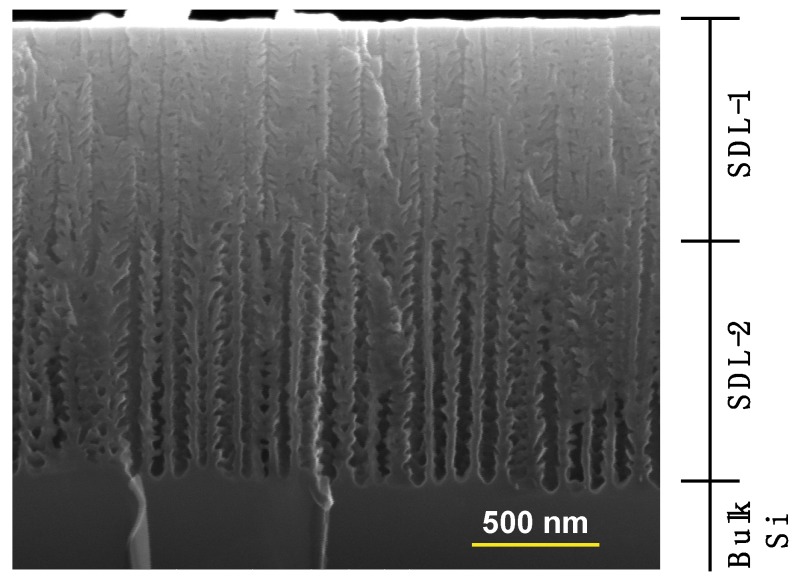
Cross-section SEM image of the sacrificial double layer (SDL) formed by the ENL process, before its dissolution. The first (SDL-1) and second (SDL-2) layers are identified by the description on the right side of the image.

As we have just described, SDL-2 is formed, using a second set of electrochemical parameters different from the first, starting from the bottom of SDL-1, as in any well-known PSi multilayer formation processes [26]. Consequently, since the HF etching solution will be present only within the already formed pores, the pore density in SDL-2 will be roughly bound to the density of SDL-1. The electrochemical parameters used for SDL-2 are chosen to obtain pores larger than those of SDL-1. The reason for this choice is that larger pores enable the control of the overall ordering of the pores by a self-ordering process, favored by a less dendritic shape of the larger pores, as shown in Figure 2 below. The self-ordering of electrochemical pore formation has already been demonstrated for larger pores in the case of alumina [27] and other materials [28] on larger scales. 

## 3. Results

An example of SDL, before the chemical dissolution, is shown in the SEM cross-section micrograph in Figure 2. SDL-1 and SDL-2 layers, indicated in the right side of the image, are clearly visible as well as the difference in diameter of the pores. In this case, the electrochemical parameters for the formation of SDL-1 and SDL-2 were HF 25% and 21% solutions with current densities of 600 and 800 mA/cm^2^, respectively. After the formation, the SDL will be dissolved using a highly concentrated aqueous NaOH solution (1M in this work), in such a way to leave an indented surface whose characteristics are determined by the features of the sacrificial double layer.

**Figure 3:**
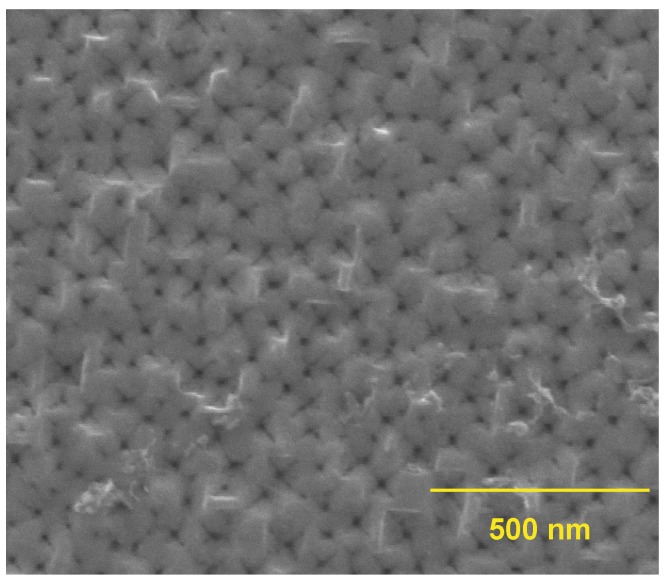
An example of the indentations on a Si surface after the ENL process.

In Figure 3, we show an example of the indentations (the seeds) remaining on the Si surface after the chemical removal of SDL, that is at the end of the ENL process. The detailed shape of the seeds depends on the SDL details and on the SDL removal chemical parameters (solution composition, etching time). The seeds are visible as small dots at the center of crosses. A cross is the image of an inverted pyramid [29] that forms after the wet chemical etch with NaOH. The inverted pyramid shape is a very good shape for a surface indentation aimed at having significant local increases of the electric field that must act as pore formation starting locations.

### 3.1. ENL Control of the Pore Density with SDL-1

**Figure 4:**
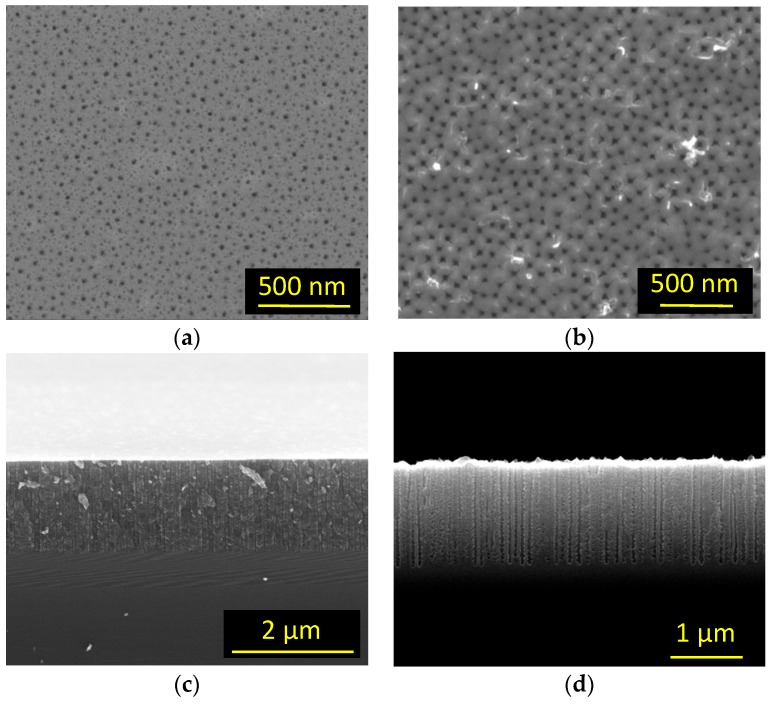
SEM images of typical PSi samples used for pore density measurements. (**a**,**b**): Top view of a standard PSi sample and of a PSi sample fabricated after ENL, respectively. (**c**,**d**): Cross-sections of the samples shown in (**a**,**b**), respectively. In the pore density measurements, several image sizes have been used for each sample to avoid possible sample inhomogeneities.

Before demonstrating the SDL-1 role, a few words have to be spent on the procedure used to measure the pore density. In Figure 4, we show the SEM image of two typical samples used for the pore density measurements. Figure 4a,b shows the top-view images of the two samples and in Figure 4c,d, the respective cross-sections. The sample of Figure 4a,c is a standard PSi layer formed with 15% HF solution and a current density of 200 mA/cm^2^, while the sample in Figure 4b,d is an L-3 layer formed using a 21% solution and a current density of 900 mA/cm^2^. In this sample, the porous layer is fabricated after the ENL process. It is important to note that in all cases the pore density was measured by counting, for each sample, the visible pores on several cross-sections (different locations and different magnifications). We obtained coherent results among different regions in each sample, indicating their homogeneity. The measure of the pore density from top surface lead instead to inconsistent results in all cases when changing image analysis parameters (that is, the parameters used to obtain a black-and-white image needed for the pores counting using ImageJ, as the color threshold for the pore size identification). This is due to the fact that, for single layers, the presence on the top surface of small openings that will not become pores affects the image treatment. This is not the case for samples after ENL, where the openings are clearly defined, as can be seen in Figure 4b,d, but we used the same approach to the pore density measurement in all samples for the sake of homogeneity. 

To demonstrate the ability of ENL to control the seed density, we decided to measure the pore density on L-3 layers. In fact, if our hypothesis on the SDL-1 role is true, the pore density of SDL-1 will first affect the one of SDL-2, then that of the seeds left on the surface after ENL, and finally, the one of L-3. Therefore, the best proof for the ENL control of seed density by SDL-1 pore density is the measure of pore density on L-3 layers fabricated using identical parameters. We then prepared several sets of L-3 layers fabricated, with identical formation parameters, after different ENL processes, so that their differences can be uniquely associated with the specific ENL parameters. Since our goal is to identify the role of SDL-1 on the L-3 pore density, the ENL formation parameters were varied from sample to sample only for SDL-1, while they were kept identical for SDL-2.

**Figure 5:**
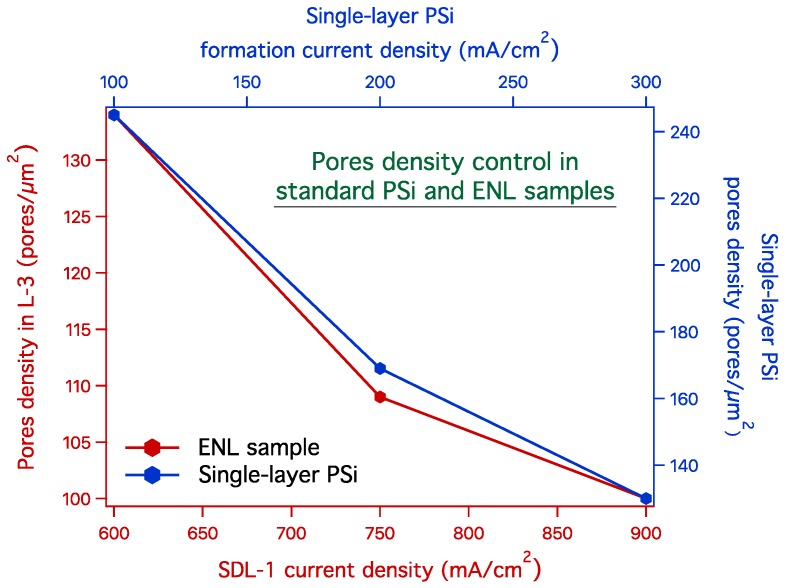
Evolution of the pore density in standard PSi samples and in samples after ENL. The graph reports the data for the pore density evolution as a function of the current density for standard PSi samples (blue curve and axes). The comparison is made with samples with ENL (red curve and axes). In the ENL double layer, the pore density was varied only in SDL-1, while the electrochemical parameters for SDL-2 were identical in all cases. The pore density for ENL samples is measured in the L-3 layer. All pore densities are measured on cross-section SEM images, as explained in the text.

The results of the pore density measurements are shown in Figure 5 where the blue curve (right and top axes) shows the evolution of pore density for a single layer of standard PSi as a function of the formation current density. As a general rule, the larger the current density, the larger the pores [22]. Therefore, the density of pores is expected to decrease with increasing formation current density, since larger pores occupy more volume and the only possible way for their formation is to reduce their number, that is to decrease the pore density. Please note that the pore density is not directly related to the samples’ porosity, which is the ratio of empty to full volumes within the pores: The porosity increases when increasing the formation current density [22]. 

The red curve in Figure 5 (left and bottom axes) shows the evolution of the L-3 pore density as a function of the SDL-1 formation current density. Since the dependence of pore density on the formation current density is a well-known fact for porous silicon and other materials as alumina [30,31], the chosen set of current densities for the blue curve Figure 5 is just one possibility among many. In analogy to the single PSi layers (blue curve), in the L-3 samples series (red curve) the SDL-1 formation parameters only differ for the formation current density. The red and blue curves show that in both cases an increase of the formation current density leads to a decrease of the pore density. However, while for the single PSi layers the variation is in the single layer where the fabrication parameters were changed, in the case of the L-3 layers the variation is produced on SDL-1 layer and measured in the L-3 layer. Therefore, these results demonstrate the control of the L-3 pore density by the first ENL layer, namely SDL-1. 

### 3.2. ENL Control of the Seed Distribution Order with SDL-2

Once the effect of SDL-1 layer is ascertained by the previously described results, the next step is the description of the effect of SDL-2 layer on the surface distribution of the seeds, and therefore, of L3 pore opening. The working principle is that of the self-ordering of the SDL-2 pores that will be induced thanks to their larger diameter and their less dendritic shape, in analogy to the previously cited works on other materials [28]. 

In analogy to the method used to evidence the effect of SDL-1, to demonstrate the ordering effect of SDL-2 we prepared several sets of three samples with identical SDL-1 and L-3 layers, changing only SDL-2 formation parameters. For SDL-2, while maintaining all other parameters identical, we increased the formation current from the first to the third sample. There are obviously many possibilities to fabricate SDL-2 pores with pore diameter larger than SDL-1. However, for the scope of this work, we chose to vary only the formation current density as a paradigm of the effect. If our hypothesis is correct, we expect that the increase of the pore diameter induced by increasing current density will lead to a more orderly pore arrangement, thanks to a self-ordering effect. Although we cannot expect a perfect arrangement, given the irregularities of the inner pore surfaces, we can expect that reducing the dendritic level and increasing the pore diameter will play a positive role in the pore ordering.

**Figure 6:**
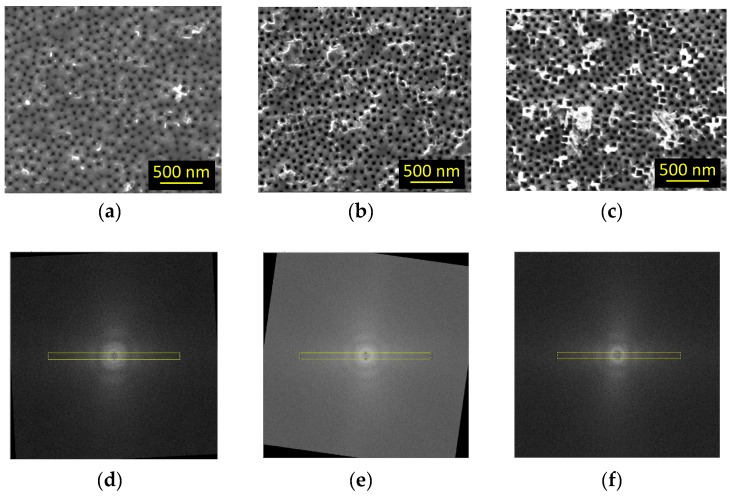
SEM images (**a**–**c**) of three PSi samples prepared with identical formation parameters after three different ENL processes. From left to right, the formation current of SDL-2 increases, while all other parameters have been kept constant. In particular, SDL-1 layers are nominally identical in all samples. On the bottom row (**d**–**f**) are shown the fast Fourier transform (FFT) of the SEM images (**a**–**c**), in the same order. The FFT has been calculated on squared areas of the respective SEM images. The yellow rectangles on the FFT images, identical for all samples, are the area over which the profiles of Figure 7 are calculated. If needed, the FFT images have been rotated to align the rectangle and the order-related fringes appearing on the FFT images, so to ensure that all images are analyzed coherently.

**Figure 7:**
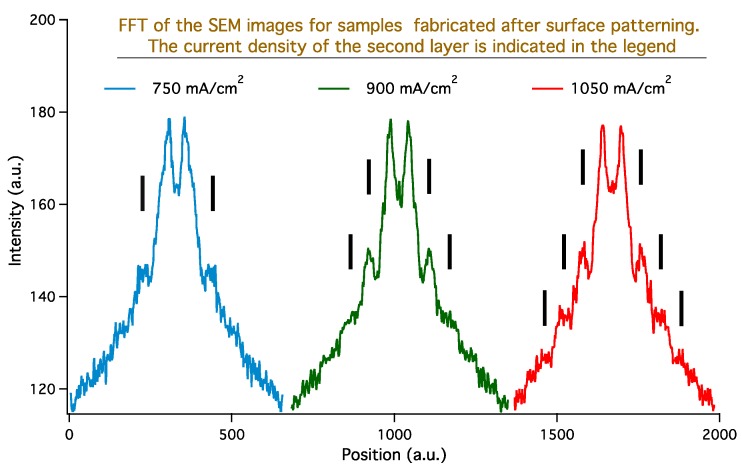
FFT profiles obtained from the FFT images in the bottom row of Figure 6. The profiles are obtained from the yellow rectangles, as described in Figure 5 caption. The order of the curves reproduces the order of the SEM images. The green and red curves have been translated along the x-axis for readability, while the blue curve is on its original position. The formation current density, with all other parameters kept identical, has been progressively increased going from the blue to the red curve. The presence of peaks, related to the pore distribution ordering, is indicated with small black vertical bars for each curve.

In Figure 6a–c, we show the SEM images of three PSi samples prepared with identical formation parameters after three different ENL processes: The SDL-2 formation current density increases from a–c, a variation that is aimed at increasing the pore distribution order in L-3. In Figure 6d–f, we report the fast Fourier transform (FFT) of the top row SEM images, in the same order. The yellow rectangle in each FFT image is the area used for the calculation of the FFT profiles that we show in Figure 7. The order of the curves corresponds to the order of the SEM images. When performing FFT, for higher number of peak couples in each curve the reproducibility of the interpore distance increases as well as the distribution order for the pore openings on the surface. In the graph, for each curve short black vertical lines indicate the presence of clearly discernible peaks. When increasing the formation current, the number of peaks visible in the FFT increases, demonstrating our hypothesis that it is possible to control the pore ordering with SDL-2 formation parameters.

### 3.3. ENL-Improved Pore Opening Homogeneity

A further interesting effect of the ENL process is the significantly improved size homogeneity of the pore openings that can be obtained thanks to the ENL seeds.

**Figure 8:**
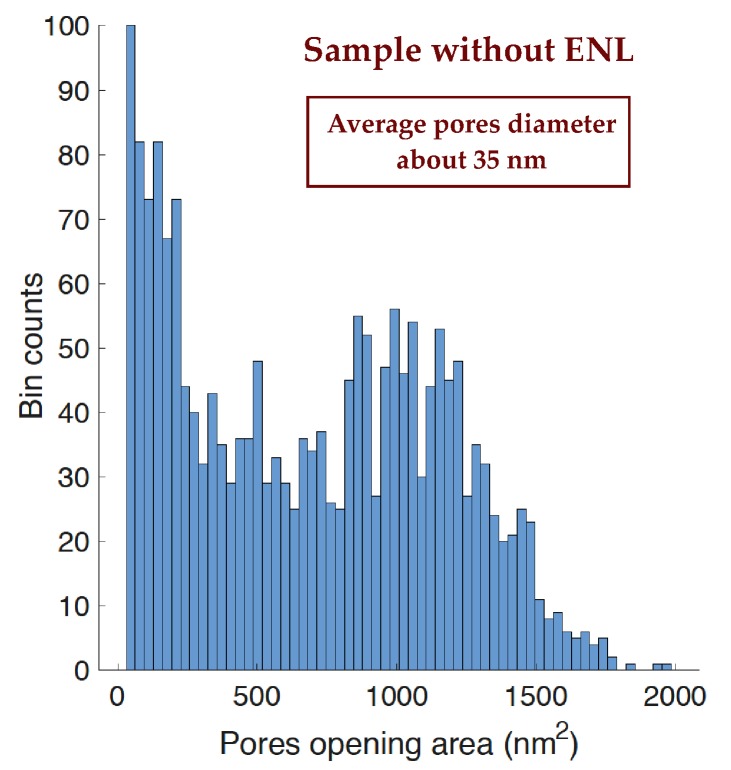
Distribution of the pore opening size for a sample without ENL before the formation.

**Figure 9:**
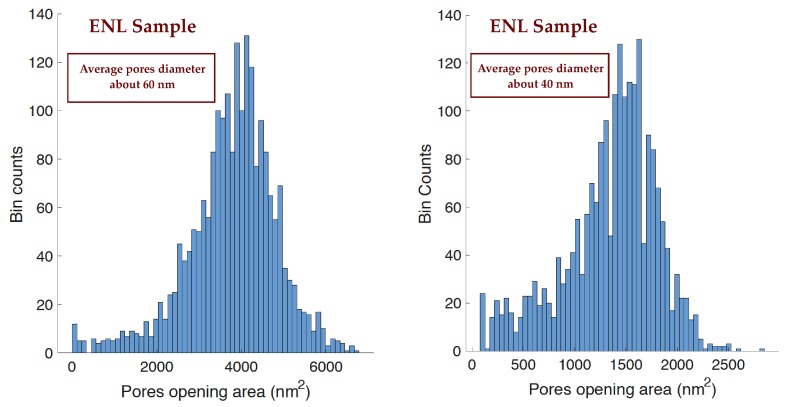
Distribution of the pore opening size for two samples that underwent the ENL process before the formation of the porous layer. The two samples have been fabricated with two different average pore diameters: About 60 nm (**left**) and about 40 nm (**right**).

The improvement in pore size homogeneity is due to the homogeneity of the seed size, that allows the fabrication of pores with equally homogeneous pore openings. The homogeneity of the distribution is substantially independent on the pore size since it is bound to the seed shape and not to the given electrochemical parameters chosen for the third layer fabrication. An example of this improved homogeneity is shown in the histograms of Figure 8 and Figure 9. Figure 8 show the histogram of the pore opening area distribution for a sample that has been prepared without the ENL process, where a clearly bimodal distribution is represented. The presence of small surface openings is clearly visible with an intense peak, whose height even surpasses that of the main peak at about 100 nm^2^ that reflects the average pore diameter in the porous layer thickness. Figure 9 shows two histograms for samples with similar ENL processes and different third layer processing. In all our measurements, the pore distribution shape after ENL is essentially the same as that observed for the two samples of Figure 9, independent of the exact ENL parameters and on the specific L-3 parameters. On the left, the histogram for a sample whose average pore diameter (from the cross section) is about 60 nm, while on the right, the histogram relative to a sample with an average pore diameter of about 40 nm, almost the same size of the sample shown in Figure 8. The difference between these two histograms with the one in Figure 8 is remarkable: No small openings-related peak, with a size distribution well centered on the average pore diameter. The average area of the pore surface openings measured in each sample from the SEM top views is in agreement with the square of the pore width measured in the corresponding SEM cross-section. This is due to the fact that the pore section is generally roughly squared. The bell shape in the histograms is clearly maintained for both pore diameters, with a presence of smaller pores very low with respect to the main peak in both cases. The improvement of the pore size homogeneity effect of the ENL process is then demonstrated: The presence of surface seeds is then a strongly limiting factor for the formation of small openings in the competitive process of the initial surface irregularities to become the host of full pores.

## 4. Conclusions

In this work, we demonstrate the feasibility of an easy and cost-effective electrochemical lithographic process at the tens-of-nm scale called electrochemical nanolithography. ENL is easily scalable to large surface and is designed to separately control the pore distribution order and density by the fabrication of a specifically designed sacrificial double layer that, after its removal, leaves an indented surface. These surface indentations act as seeds for the formation of a new porous layer whose parameters can be chosen by the experimenter, but whose pore distribution and density are bound by the ENL seed distribution and density.

## 5. Patents

A patent application has been submitted on the electrochemical nanolithography process, based on the idea described in this work. The patent application number is in the reference list [24].
